# Prediction models for drug-induced hepatotoxicity by using weighted molecular fingerprints

**DOI:** 10.1186/s12859-017-1638-4

**Published:** 2017-05-31

**Authors:** Eunyoung Kim, Hojung Nam

**Affiliations:** School of Electrical Engineering and Computer Science, Gwangju Institute of Science and Technology (GIST), Buk-gu, Gwangju, 61005 Republic of Korea

**Keywords:** Drug toxicity prediction, Drug-induced liver injury, Machine learning, Data mining

## Abstract

**Background:**

Drug-induced liver injury (DILI) is a critical issue in drug development because DILI causes failures in clinical trials and the withdrawal of approved drugs from the market. There have been many attempts to predict the risk of DILI based on in vivo and *in silico* identification of hepatotoxic compounds. In the current study, we propose the *in silico* prediction model predicting DILI using weighted molecular fingerprints.

**Results:**

In this study, we used 881 bits of molecular fingerprint and used as features describing presence or absence of each substructure of compounds. Then, the Bayesian probability of each substructure was calculated and labeled (positive or negative for DILI), and a weighted fingerprint was determined from the ratio of DILI-positive to DILI-negative probability values. Using weighted fingerprint features, the prediction models were trained and evaluated with the Random Forest (RF) and Support Vector Machine (SVM) algorithms. The constructed models yielded accuracies of 73.8% and 72.6%, AUCs of 0.791 and 0.768 in cross-validation. In independent tests, models achieved accuracies of 60.1% and 61.1% for RF and SVM, respectively. The results validated that weighted features helped increase overall performance of prediction models. The constructed models were further applied to the prediction of natural compounds in herbs to identify DILI potential, and 13,996 unique herbal compounds were predicted as DILI-positive with the SVM model.

**Conclusions:**

The prediction models with weighted features increased the performance compared to non-weighted models. Moreover, we predicted the DILI potential of herbs with the best performed model, and the prediction results suggest that many herbal compounds could have potential to be DILI. We can thus infer that taking natural products without detailed references about the relevant pathways may be dangerous. Considering the frequency of use of compounds in natural herbs and their increased application in drug development, DILI labeling would be very important.

**Electronic supplementary material:**

The online version of this article (doi:10.1186/s12859-017-1638-4) contains supplementary material, which is available to authorized users.

## Background

As the leading cause of development failure in clinical trials and withdrawal of drugs from the market, drug-induced liver injury (DILI) is one of the most important factor in drug development [1]. The severe adverse effects of DILI, which include acute liver failure and jaundice, must be considered in drug development. The toxicity of these drugs is attributable to their conversion in the liver to highly reactive metabolites that cause organ damage [2–4]. However, determining DILI potential is a very challenging task, primarily because animal studies do not efficiently predict DILI potential in human. For example, in a phase II clinical trial, acute liver toxicity induced by fialuridine led to the deaths of five subjects, in contrast to its safe use in animal studies [5]. In a study of 221 pharmaceutical products, the rate of concordance of hepatotoxicity in humans and animals was low, approximately 55%, whereas the rate of concordance was much higher in other target organs, including the hematological (91%), gastrointestinal (85%), and the cardiovascular (80%) systems [6]. In addition, clinical features or laboratory tests for predicting DILI potential have not been identified [7, 8]. Moreover, the statistical power of clinical trials is insufficient. Severe idiosyncratic hepatotoxicity occurs at very low frequency, and patient samples in clinical trials number only in the thousands. Due to this low statistical power, even well-controlled clinical trials can fail to predict DILI.

To overcome these problems, many researchers have sought to evaluate the toxicity of compounds in vitro and/or in vivo. However, considering the number of compounds, this approach is time-consuming and costly, and thus there has been much effort to develop prediction models to determine if a compound could cause liver toxicity. Computational modeling approaches have been adopted by pharmaceutical companies to help evaluate the efficacy, toxicity, and metabolism of pharmaceutical ingredients [9]. In the early stages of the development of prediction models, the predictive power of the constructed models was not satisfactory, and models often relied on experimental data for better performance. Some researchers used molecular signatures, such as for alanine transaminase (ALT), aspartate aminotransferase (AST), and alkaline phosphatase (ALP), all of which are commonly assessed in the diagnostic evaluation of hepatocellular damage [10]. In more recent years, machine-learning algorithms for prediction models have also been developed to obtain better predictions [11, 12]. However, experimental data are limited utility in constructing prediction models. Therefore, several researchers have focused on computational predictions using compound properties and structural characteristics. Greene et al. developed structure-activity relationships for potentially hepatotoxic compounds [13]. Compounds were categorized into four classes associated with hepatotoxicity: no evidence, weak evidence, animal hepatotoxicity and human hepatotoxicity. The resultant hepatotoxicity alerts yielded a concordance of 56%, a specificity of 73%, and a sensitivity of 46%. Ekins et al. built a classification model based on the Bayesian modeling method with molecular descriptors and fingerprint descriptors [14]. The evaluation of the classifier demonstrated a concordance of 60% for internal validation and 64% for external validation. Rodgers et al. also developed a quantitative structure-activity relationship (QSAR) model using liver adverse effects of drugs (AEDs) as a dataset. They used information on enzyme markers of hepatotoxicity, but these markers can fluctuate due to other factors throughout the day [15]. Moreover, Huang et al. developed a prediction model based on QSAR using a variety of descriptors including fingerprints. Their model performed well with an accuracy of 79.1% in internal validation. They further predicted the potential hepatotoxicity of Traditional Chinese Medicines [16]. Zhang et al. also developed an *in silico* prediction model for DILI. They used three different fingerprints and five machine-learning algorithms and obtained a concordance of 66% using the Support Vector Machine algorithm and FP4 fingerprint, in addition to identifying important substructure patterns related to liver toxicity [17]. Despite these extensive efforts to predict DILI, there are no standard QSAR models for DILI, in contrast to the availability of QSAR models for mutagens. Moreover, less is known about the substructures that are significantly associated with DILI [18–20].

**Fig. 1:**
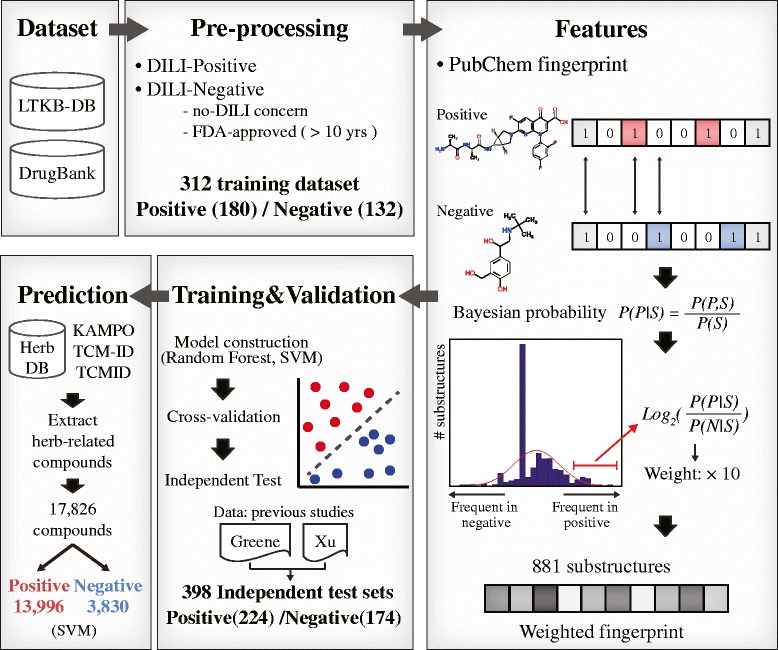
Overview of prediction model construction

Thus, in this study, we focused on improving DILI prediction models using Bayesian weighted substructures and identifying frequently appearing substructures that might be key for DILI (Fig. 1). First, datasets from the Liver Toxicity Knowledge Base (LTKB) and the DrugBank database were obtained and pre-processed [21]. We then extracted substructure feature information from 312 compounds. The weighted features were obtained from the calculation of the Bayesian probability for each substructure represented in a compound fingerprint. The prediction models were trained by two algorithms and evaluated with an independent test set of unseen 398 compounds. Finally, the constructed models were used to predict the hepatotoxic potential of herb-related compounds from herb databases. Moreover, several frequent substructures related to DILI-positive compounds were reported as alerts.

## Methods

### Data preparation

The Liver Toxicity Knowledge Base Benchmark Dataset (LTKB-BD) and the DrugBank database were used as training datasets. LTKB-BD is a benchmark dataset provided by the National Center for Toxicological Research (NCTR), U.S. FDA [21, 22]. This dataset contains a list of drugs with DILI potential in humans in accordance with FDA-approved prescription drug labels. Drugs in the dataset are categorized into one of three groups based on their description and severity: most-DILI-concern, less-DILI-concern, and no-DILI-concern. Drugs with a black box warning of hepatotoxicity or that were withdrawn from the market were classified into the most-DILI-concern category. The drugs in that class were labeled due to their fatal hepatotoxicity, including liver necrosis, jaundice, and acute liver failure. The less-DILI-concern drugs included those with moderate DILI warnings, and drugs without any DILI indication were classified as no-DILI-concern drugs. In this study, we began by labeling 222 DILI-concern drugs and 65 no-DILI-concern drugs from the LTKB-BD as positive and negative, respectively. We then retrieved simplified molecular-input line-entry system (SMILES) information using ChemSpider python API by name matching [23, 24]. The SMILES information was further used to obtain molecular fingerprints for use as features in model training and construction. We selected only one-matched compounds for higher confidence because ChemSpider API offers a partial matching service. Finally, we obtained 180 positive and 53 negative compounds.

Moreover, we retrieved additional negative data from the DrugBank database to balance the data size. From the DrugBank database, we extracted FDA-approved drugs, with a focus on drugs approved for more than 10 years. The database provides a ‘started-market-date’ and an ‘ended-market-date’, and thus we set the limits to ‘2006’ for the started-market-date and to ‘none’ for the ended-market-date. We again queried ChemSpider API to obtain the SMILES information for these drugs, and we removed the drugs overlapping with the LTKB dataset by comparing the SMILES information. Finally, we identified 79 negative compounds from the DrugBank database. In total, 180 positive compounds and 132 negative compounds were used as the training dataset as listed in Table 1.

**Table 1:** The number of compounds used in training and the independent test

Datasets		DILI-positive	DILI-negative	Total
Training	LTKB	180	53	312
	DrugBank	-	79	
Independent test	Green & Xu	224	174	398

### Molecular fingerprints

Molecular fingerprints are a representation of the structure of a compound. Fingerprints are widely used in chemical informatics because they consist of bitstrings, which facilitate molecule comparisons. Each bit of a fingerprint represents a specific substructure of a molecule, and the annotation of the substructure depends on the type of fingerprint. In the current study, we used PubChem fingerprints (ftp://ftp.ncbi.nlm.nih.gov/pubchem/specifications/pubchem_fingerprints.pdf), which have a length of 881 bits. Each bit represents the presence of an element, the count of a ring system, the atom pairs, the atom’s nearest neighbors, and the SMARTS patterns. The PubChem fingerprint was chosen for substructure reporting in the present study because it describes the structure of a molecule in detail with a long bit-vector. To retrieve fingerprint information, we used the PaDEL-Descriptor, which is software used to calculate molecular descriptors including 1D, 2D, and 3D descriptors and 12 types of fingerprints for the PubChem fingerprint [25]. The software can be downloaded online and supports a graphical interface.

### Bayesian theory for feature weight calculation

A molecular fingerprint is a binary vector and thus is composed of zeros and ones. The fingerprint indicates the presence of a substructure in a molecule. In this study, we focused on substructure information in DILI-positive compounds, and therefore, we used Bayesian theory to identify frequent substructures in DILI-positive compounds that might cause hepatotoxicity. First, we calculated the probability that a compound was DILI-positive/negative given that a structure was present/absent (Formula 1), where *P* and *N* each represents positive and negative label, and *S* indicates a substructure.

1 $$ P\left( P\Big| S\right)=\frac{P\left( P, S\right)}{P(S)}=\frac{P\left( S\Big| P\right) P(P)}{P\left( S\Big| P\right) P(P)+ P\left( S\Big| N\right) P(N)} $$

However, if we calculate the Bayesian probability as in the equation above, a substructure will have a probability value of zero if it is absent from both positive and negative compounds. A zero probability does not indicate that a substructure is always absent in either case. If we increase the size of the dataset, those bits might appear. Therefore, to avoid zero probabilities, we used Laplace smoothing, which is a technique that pretends we observed every outcome k extra times (Formula 2).

2 $$ {P}_{LAP, k}(x)=\frac{c(x)+ k}{N+ k\left| X\right|},\ {P}_{LAP, k}\left( x\Big| y\right)=\frac{c\left( x, y\right)+ k}{c(y)+ k\left| X\right|} $$

We then calculated the log odds ratio for each substructure (Formula 3).

3 $$ L o{g}_2\left(\frac{P\left( P\Big| S\right)}{P\left( N\Big| S\right)}\right) $$

**Fig. 2:**
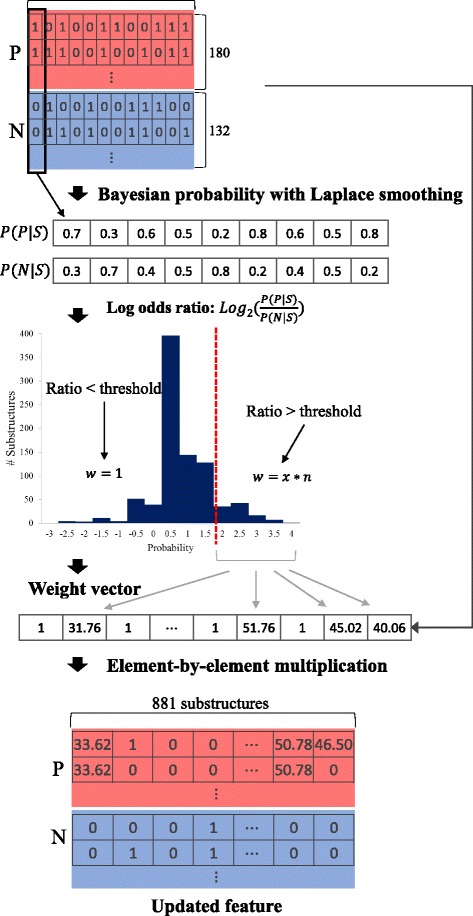
The process of feature weight calculation. First, the Bayesian probabilities for each substructure were calculated. Then, substructures selected based on a log odds ratio threshold were weighted, while others remained binary. When calculating the weight vector, the feature values (x) of selected substructures were amplified by a user parameter *n*. The constructed weight vector was then multiplied with the original feature matrix

If the ratio value of a substructure is high, it means that the substructure appeared more frequently in DILI-positive compounds. We then set the threshold to give weight using the log odds ratio values. The values of the selected substructures that were greater than the threshold were weighted by multiplying and amplifying the original odds ratio by *n* in Fig. 2. By contrast, the substructures with odds ratio below the threshold received a weight value of one. Here, we only gave weight to high log odds ratios because we wanted to predict DILI-positive compounds, which are toxic and therefore more critical to predict than negative compounds. The calculated weight vector was then multiplied element-by-element to the original fingerprint. The overall process of weight calculation is illustrated in Fig. 2.

The Random Forest (RF) and the Support Vector Machine (SVM) algorithms were used to construct the classification and prediction model. The RF algorithm is an ensemble learning algorithm that operates by constructing a large number of decision trees and collecting them. When it devises a prediction, it runs a new input for every decision tree and votes on how it is to be classified. The main advantage of the RF algorithm is that it avoids overfitting problems, which occur frequently when dealing with a small dataset. The implementation of the algorithm is found in MATLAB Statistics and Machine Learning Toolbox (MATLAB and Statistics Toolbox Release 201#, The MathWorks, Inc., Natick, Massachusetts, United States). The TreeBagger function was used for the RF algorithm. SVMs are among the most popular supervised machine-learning algorithms for pattern recognition and are also used for classification. SVM constructs a hyperplane that is used for classification using specified training examples, each including a category label. The constructed model can then be used to predict the DILI potential of a new drug. The implementation of the SVM we used is A Library for Support Vector Machines (LIBSVM) [26]. When training a model, we used similarity matrices calculated using the Tanimoto coefficient, a similarity metric that uses the ratio of the intersecting set to the union set because the constructed space would be very high-dimensional with 881 features. The use of similarity matrices reduces the dimensions to the data size.

When training the models, we performed 10-fold cross-validation, which divides the training dataset into ten subsamples. Nine subsamples are used for training, and one subsample is used for testing. We constructed each model with different thresholds and multiplication numbers, and we compared the performances to select the best model for prediction.

### Independent test

The data from previous studies were used for further evaluation. We collected the independent test set from two studies: Greene et al. and Xu et al. [13, 27]. Greene’s dataset was categorized into four groups: HH (evidence of human hepatotoxicity); NE (no evidence of hepatotoxicity in any species); WE (weak evidence of human hepatotoxicity); and AH (evidence for animal hepatotoxicity but not tested in humans). To use strict data, we used the compounds in the HH and NE categories as positive and negative, respectively. After combining the two datasets, we pre-processed the resultant dataset in the same manner as the training set. The SMILES information was retrieved from ChemSpider and was used to eliminate duplicates from the training set and eliminate label contradictions between the two sets. In total, we obtained 398 compounds, including 224 positive and 174 negative.

### Prediction of natural products

The constructed classification model was then applied to predict the potential hepatotoxicity of natural products. We collected herbal compound information from the TCMID, TCM-ID, and KAMPO databases [28–30], all of which contain information about the efficacy of herbs and their constituent compounds. The natural product dataset was also standardized by ChemSpider, and a fingerprint was obtained. Fingerprints were not able to be retrieved for a few compounds, primarily very complex, large molecules with a mass greater than 1000 Da. These compounds were excluded, resulting in a final total of 17,826 compounds.

## Results

### Frequent substructures in hepatotoxic compounds

One of the main purposes of this research was to identify important substructures in DILI-positive compounds. The frequently appearing substructures can be inferred from the weighted substructures. We first calculated the probabilities of each substructure to be in positive and negative labeled compounds respectively. Then with the log odds ratio of positive to negative we selected substructures to be weighted. We determined the weighted substructures by high log odds ratio values, since we focused on substructures which are frequent in DILI-positive compounds. With a log odds ratio threshold of 2.5, we identified 24 substructures.The following substructures with other various threshold values are described in Additional file 1: Table S1–S3.

### Model performance

**Fig. 3:**
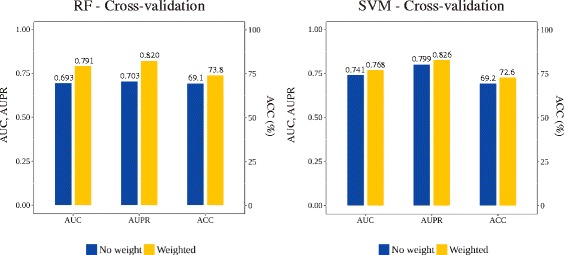
Performance of the models in cross-validation. Performance in both RF and SVM increased with weighted features

We compared the model without weighted features to the model with weighted features to assess whether giving weights to the frequently appearing substructures affected performance. As shown in Fig. 3, models with weighted features performed better in both algorithms. Although the RF model previously performed poorly, with the weighted feature, the AUC, AUPR, and accuracy increased significantly to 0.79, 0.82, and 74%, respectively. Likewise, the SVM performance also increased, although models without features were already classified quite well. The AUC, AUPR, and accuracy values were 0.77, 0.83, and 73%, respectively. All models with different thresholds and multiplication numbers were compared. The RF model performed best with a threshold of 1.5 and a multiplication number of 15, and the SVM model performed best with a threshold of 2 and multiplication number of 15. A performance comparison using different thresholds can be found in Additional file 2: Figure S1–S2.

**Fig. 4:**
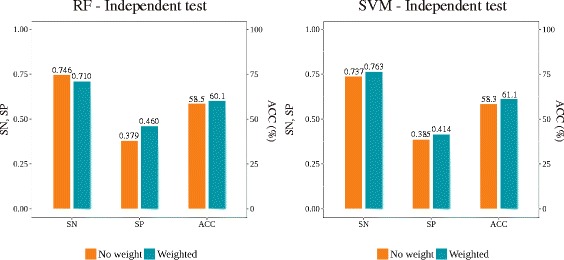
Performance of the models in the independent test. The gap between sensitivity and specificity decreased and the accuracy increased with weighted features in both models

Furthermore, we compared the performance of the constructed models in an independent test to evaluate the performance with unseen data set. Figure 4 shows the increased performance with the weighted features. Although the sensitivities were high in the non-weighted models, the specificities were very poor. Using the weighted feature, the specificity of both models increased to greater than 0.4, and the overall accuracy values increased slightly.

**Fig. 5:**
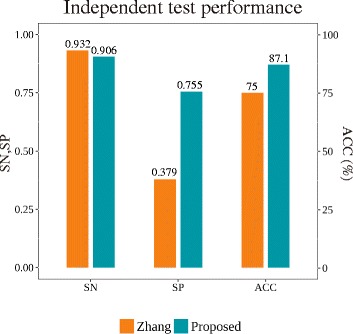
Performance comparison between the previous study and the proposed method. Our method increased the performance overall compared with that reported by Zhang. In particular, the specificity increased dramatically, although the sensitivity decreased slightly

We implemented a model from Zhang’s study for further performance comparison. They developed prediction models with various fingerprints and machine-learning algorithms. We constructed an SVM model with the dataset provided by Zhang et al. using FP4 fingerprints and applied our proposed feature weight calculation method. Our method increased the accuracy from 75% to 87% (Fig. 5). Although the sensitivity decreased slightly, the specificity increased dramatically from 0.379 to 0.755, indicating that our method performs well in predicting both negative and positive compounds. As a more precise comparison, we randomly selected 59 positive and 29 negative compounds from the LTKB dataset a hundred times, and our method resulted in a higher average accuracy of 86.4%. This result indicates that our method exhibits superior classification and prediction of DILI compounds under the same conditions.

### Prediction of hepatotoxic compounds in natural products

**Fig. 6:**
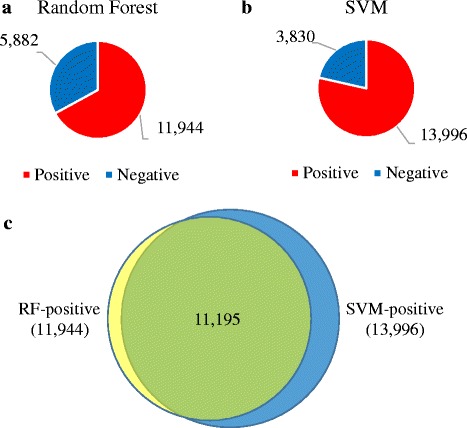
The proportion of predicted compounds in herbs. **a** RF predicted 67% of compounds as DILI-positive. **b** SVM predicted 79% of compounds as DILI-positive. **c** The number of overlapping compounds predicted by the two algorithms

The hepatotoxic potential of the herb-related compounds was predicted using the constructed models. Since the parameters and algorithms in each model vary, the results differed slightly, but the models predicted that more than 60% of compounds in natural products have hepatotoxic potential. RF predicted 11,944 compounds as hepatotoxic, whereas SVM predicted 13,996 compounds as DILI-positive. Although the two prediction models yielded different outcomes, the predicted positive compounds greatly overlapped, as shown in Fig. 6.

## Discussion

In the current study, we calculated the weighted feature using Bayesian theory and constructed DILI prediction models using the updated feature with two algorithms: RF and SVM. When calculating the weight vector, we focused on giving weight to those features that appeared more frequently in DILI-positive compounds than in DILI-negative compounds because it is more important to identify hepatotoxic compounds that might cause critical adverse reactions when developed into drugs. Therefore, we set a cutoff to select the substructures to be weighted by their log odds ratio values. The threshold ranged from 0.5 to 2.5 and resulted in different performances. With an excessively low threshold, the number of weighted substructures was too large, causing the overall values of the weight vector to increase without differentiating specific substructures and, consequently, poor model performance. By contrast, the use of an excessively high threshold would weight too few substructures, resulting in a decrease of performance. The parameter multiplied with the selected substructure also affected the performance, but the effect was not significant. This result indicates that amplification of values is important but that the degree of amplification does not significantly affect model performance.

Both constructed models resulted in good performance in cross-validation considering AUC and accuracy; however, the accuracy of the independent test slightly decreased compared to the results of cross-validation. The low accuracy was due to low specificity, indicating that the model tends to predict more compounds as positive than it predicts as negative. This problem occurred because we focused on predicting DILI-positive compounds by weighing the related substructures and used a sensitivity threshold of 0.8, which could be relatively high. Because it is safer to predict negative compounds as positive (classifying nontoxic compounds as toxic) than to classify toxic compounds as nontoxic, we did not lower the threshold but attempted to reduce the gap between sensitivity and specificity using a weighted feature. This approach helped increase the accuracy. Although the increase in accuracy was not dramatic, the model classified the independent test set more precisely, positive to positive and negative to negative. The results also demonstrated that the weighted substructures affected the prediction of DILI-positive compounds.

In this study, we also determined frequently occurring substructures in DILI-positive compounds. Although the substructures with the highest probability are general, as the threshold lowers, more details in the SMARTS patterns can be observed. We obtained general structures because of the characteristic of PubChem fingerprints, which divide a structure into lower levels.

**Fig. 7:**
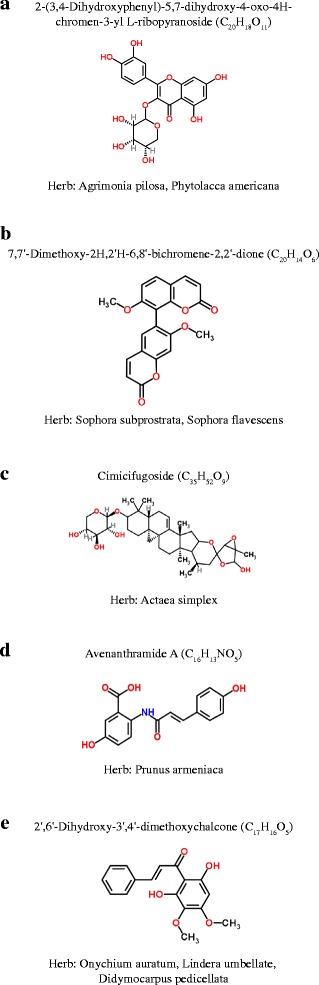
Examples of predicted DILI-positive compounds and related herbs. Each compound is represented with its name, formula, structure and its related herbs. Each compound is related to following herbs - **a** Agrimonia pilosa, Phytolacca americana **b** Sophora subprostrata, Sophora flavescens **c** Actaea simplex **d** Prunus armeniaca **e** Onychium auratum, Lindera umbellate, Didymocarpus pedicellata

The prediction of the DILI potential of natural products indicated that many compounds are related to drug-induced hepatotoxicity (Fig. 6). If compounds found in the intersection of the predicted results from the two algorithms are considered highly hepatotoxic, 63% of natural products from the herb databases have the potential to cause liver toxicity. We reported five compounds of 11,195 as examples in Fig. 7, including the names, structures, and related herbs that contain each compound.

## Conclusions

We introduced a DILI prediction model with weighted features. The weighted features were calculated using Bayesian probability giving information of frequency of each substructure in DILI-positive and DILI-negative compounds. As a result, the weighted features increased the model performance in both cross-validation and independent test with unseen dataset. Moreover, we applied the constructed model to prediction of DILI potential in herbs. The results show that large number of predicted positive compounds indicates that even compounds found in nature can be toxic and harmful to the human body. This finding is important because some people in Eastern countries rely on herbal medicine and believe it is safer than taking general drugs. However, natural products are not always beneficial to health. In addition, natural products have come to the forefront in drug discovery and development. Therefore, herbs that are used as home remedies or that are under development must be carefully administered, considering their toxic effects on the human body. In addition, we listed frequent substructures in DILI-positive compounds to facilitate drug screening in less time and at lower cost.

As an additional approach, we can improve the prediction models using structural information other than two-dimensional structural information. The frequent substructures we reported here based on the fingerprint annotation can be further developed to aid the identification of toxicophores using neural networks.

## Additional files

Additional file 1: Table S1. Description of frequent appearing substructures in DILI-positive compounds (Log odds ratio: 2.5). **Table S2.** Description of frequent appearing substructures in DILI-positive compounds (Log odds ratio: 2). **Table S3** Description of frequent appearing substructures in DILI-positive compounds (Log odds ratio: 2). (PDF 55 kb)

Additional file 2: Figure S1. Performance change by different cutoff. **Figure S2.** Performance change by weight values. (PDF 326 kb)
