# Supplementary material for: Prediction models for drug-induced hepatotoxicity by using weighted molecular fingerprints
Source: BMC Bioinformatics. 2017 May 31;18(Suppl 7):227. doi: 10.1186/s12859-017-1638-4 (PMC5471939; doi:10.1186/s12859-017-1638-4)
Supplement: Supplementary file 1 — Description of frequent appearing substructures in DILI-positive compounds (Log odds ratio: 2.5). Table S2. Description of frequent appearing substructures in DILI-positive compounds (Log odds ratio: 2). Table S3 Description of frequent appearing substructures in DILI-positive compounds (Log odds ratio: 2). (PDF 55 kb) [file 12859_2017_1638_MOESM1_ESM.pdf]

Supplementary Table 1. Description of frequent appearing substructures in DILI-positive compounds (Log odds ratio: 2.5)

Fingerprint #	description
44	>= 2 Br
117	>= 1 saturated or aromatic nitrogen-containing ring size 3
298	C-I
328	C(~Br)(~C)(~C)
329	C(~Br)(~H)
331	C(~Br)(:N)
365	C(~H)(~N)
422	N=N
423	N=O
439	C(-C)(-N)(=O)
455	N(-O)(=O)
474	S:C:C:N
475	S-C=N-C
476	C-O-C=C
490	C-C-C=C
501	Cl-C:C-C
509	Br-C:C-C
510	[#1]-N-N-[#1]
514	O-N-C-C
555	N=C-C=C
559	O-C-N-[#1]
622	O=C-O-C:C
671	O=C-C=C-C
676	N#C-C-C-C

Supplementary Table 2. Description of frequent appearing substructures in DILI-positive compounds (Log odds ratio: 2)

Fingerprint #	description
44	>= 2 Br
117	>= 1 saturated or aromatic nitrogen-containing ring size 3
130	>= 1 saturated or aromatic carbon-only ring size 4
133	>= 1 unsaturated non-aromatic carbon-only ring size 4
215	>= 1 saturated or aromatic nitrogen-containing ring size 7
298	C-I
301	N-O
302	N-F
328	C(~Br)(~C)(~C)
329	C(~Br)(~H)
331	C(~Br)(:N)

360	C(~Cl)(~Cl)
365	C(~H)(~N)
379	C(~N)(:N)
384	C(:C)(:C)
389	C(:N)(:N)
396	N(~C)(:C)
410	P(~C)(~C)
413	S(~C)(~H)
422	N=N
423	N=O
424	N=P
439	C(-C)(-N)(=O)
455	N(-O)(=O)
468	O=S-C-N
474	S:C:C:N
475	S-C=N-C
476	C-O-C=C
478	S-C=N-[#1]
481	O-S-C:C
487	N-C=N-C
490	C-C-C=C
497	O-C:C:N
501	Cl-C:C-C
508	S=C-N-C
509	Br-C:C-C
510	[#1]-N-N-[#1]
514	O-N-C-C
516	[#1]-C=C-[#1]
518	O=C-N-N
519	N=C-N-C
527	C:C:N-[#1]
530	N:C-C:C
532	S-C:C-[#1]
533	S-C:C-N
534	S-C:C-O
555	N=C-C=C
559	O-C-N-[#1]
587	N-C:C-O-[#1]
617	C-C-C-O-[#1]
622	O=C-O-C:C
630	O-C:C-O-C

645	O=C-N-C-C
659	O-C-C-N-C
671	O=C-C=C-C
676	N#C-C-C-C
719	Oc1ccc(O)cc1
723	Oc1ccc(Br)cc1
729	Nc1ccc(Cl)cc1
751	Nc1cc(Br)ccc1
774	Clc1c(Br)cccc1
782	OC1CCC(O)CC1
786	OC1CCC(Br)CC1
792	NC1CCC(Cl)CC1
814	NC1CC(Br)CCC1
837	ClC1C(Br)CCCC1

Supplementary Table 3. Description of frequent appearing substructures in DILI-positive compounds (Log odds ratio: 2)

Fingerprint #	description
24	>= 2 F
44	>= 2 Br
117	>= 1 saturated or aromatic nitrogen-containing ring size 3
130	>= 1 saturated or aromatic carbon-only ring size 4
132	>= 1 saturated or aromatic heteroatom-containing ring size 4
133	>= 1 unsaturated non-aromatic carbon-only ring size 4
148	>= 1 unsaturated non-aromatic nitrogen-containing ring size 5
149	>= 1 unsaturated non-aromatic heteroatom-containing ring size 5
190	>= 2 unsaturated non-aromatic nitrogen-containing ring size 6
214	>= 1 saturated or aromatic carbon-only ring size 7
215	>= 1 saturated or aromatic nitrogen-containing ring size 7
219	>= 1 unsaturated non-aromatic heteroatom-containing ring size 7
220	>= 2 any ring size 7
257	>= 2 aromatic rings
288	C-Na
294	C-Cl
298	C-I
301	N-O
302	N-F
328	C(~Br)(~C)(~C)
329	C(~Br)(~H)
331	C(~Br)(:N)
354	C(~C)(~Si)

360	C(~Cl)(~Cl)
365	C(~H)(~N)
379	C(~N)(:N)
380	C(~O)(~O)
384	C(:C)(:C)
389	C(:N)(:N)
396	N(~C)(:C)
402	N(~O)(:O)
405	O(~C)(~C)
410	P(~C)(~C)
413	S(~C)(~H)
422	N=N
423	N=O
424	N=P
439	C(-C)(-N)(=O)
455	N(-O)(=O)
456	P(-O)(=O)
468	O=S-C-N
472	C:N:C-C
474	S:C:C:N
475	S-C=N-C
476	C-O-C=C
478	S-C=N-[#1]
481	O-S-C:C
485	N:C:C:N
487	N-C=N-C
488	N-C=N-[#1]
490	C-C-C=C
497	O-C:C:N
501	Cl-C:C-C
504	N:C:N-C
508	S=C-N-C
509	Br-C:C-C
510	[#1]-N-N-[#1]
514	O-N-C-C
515	N-N-C-C
516	[#1]-C=C-[#1]
518	O=C-N-N
519	N=C-N-C
527	C:C:N-[#1]
530	N:C-C:C

532	S-C:C-[#1]
533	S-C:C-N
534	S-C:C-O
555	N=C-C=C
559	O-C-N-[#1]
584	C:C-C=C-C
587	N-C:C-O-[#1]
602	O=C-C-N-C
606	O-C:C-C-C
617	C-C-C-O-[#1]
622	O=C-O-C:C
625	O=C-C:C-O
630	O-C:C-O-C
645	O=C-N-C-C
659	O-C-C-N-C
670	Br-C:C:C-C
671	O=C-C=C-C
674	N-C-N-C:C
676	N#C-C-C-C
716	Cc1ccc(N)cc1
719	Oc1ccc(O)cc1
723	Oc1ccc(Br)cc1
729	Nc1ccc(Cl)cc1
740	Oc1cc(O)ccc1
751	Nc1cc(Br)ccc1
758	Cc1c(N)cccc1
766	Sc1c(S)cccc1
774	Clc1c(Br)cccc1
779	CC1CCC(N)CC1
782	OC1CCC(O)CC1
786	OC1CCC(Br)CC1
792	NC1CCC(Cl)CC1
803	OC1CC(O)CCC1
814	NC1CC(Br)CCC1
821	CC1C(N)CCCC1
829	SC1C(S)CCCC1
837	ClC1C(Br)CCCC1
