# Supplementary material for: Prediction models for drug-induced hepatotoxicity by using weighted molecular fingerprints
Source: BMC Bioinformatics. 2017 May 31;18(Suppl 7):227. doi: 10.1186/s12859-017-1638-4 (PMC5471939; doi:10.1186/s12859-017-1638-4)
Supplement: Supplementary file 2 — Performance change by different cutoff. Figure S2. Performance change by weight values. (PDF 326 kb) [file 12859_2017_1638_MOESM2_ESM.pdf]

Supplementary Figure 1. Performance change by different cutoff

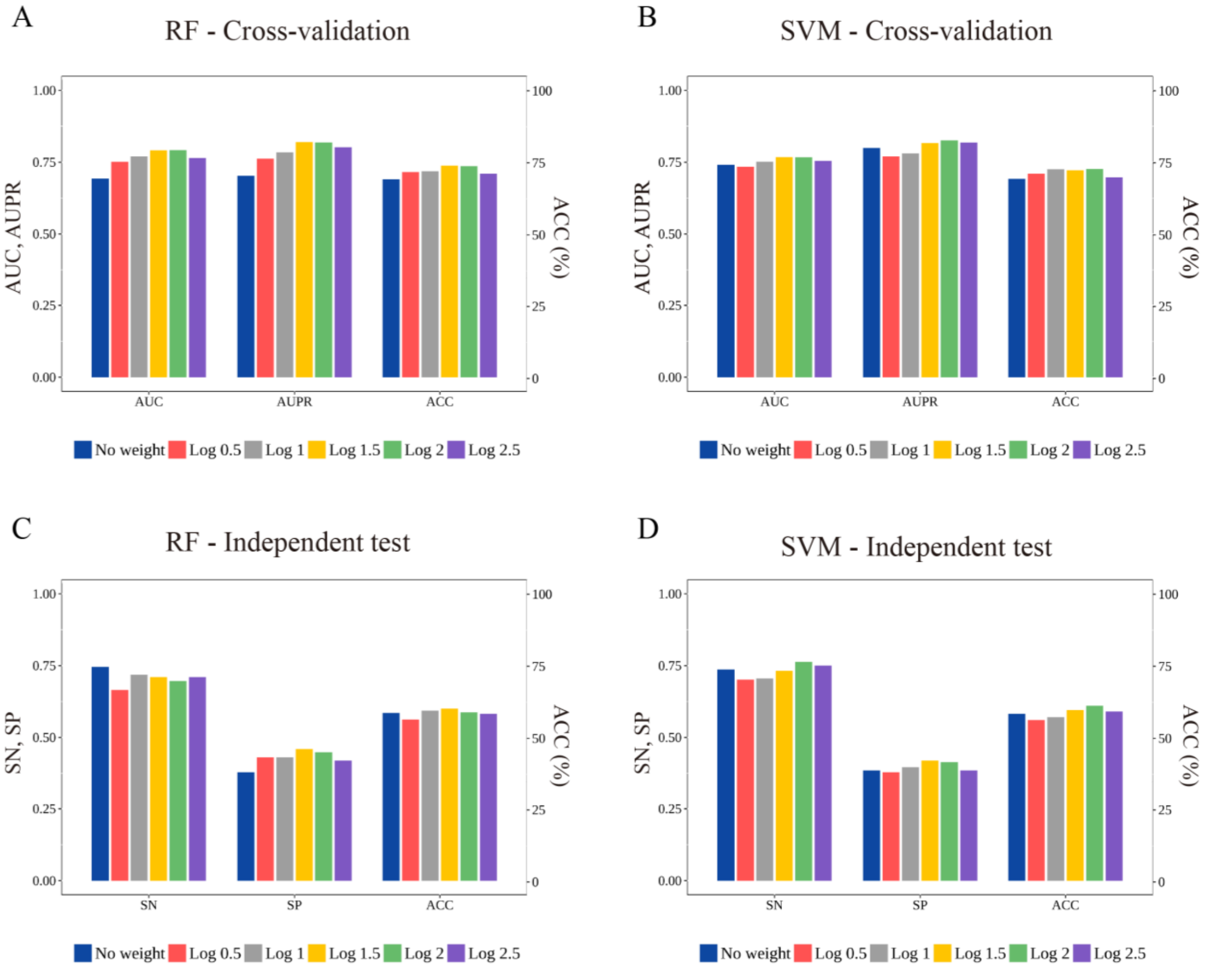

A. Performance change in cross-validation by different cutoff (Random Forest). B. Performance change in cross-validation by different cutoff (SVM). C. Performance change in independent test by different cutoff (Random Forest). D. Performance change in independent test by different cutoff (SVM). The cutoff value ranges from 0.5 to 2.5.

Supplementary Figure 2. Performance change by weight values

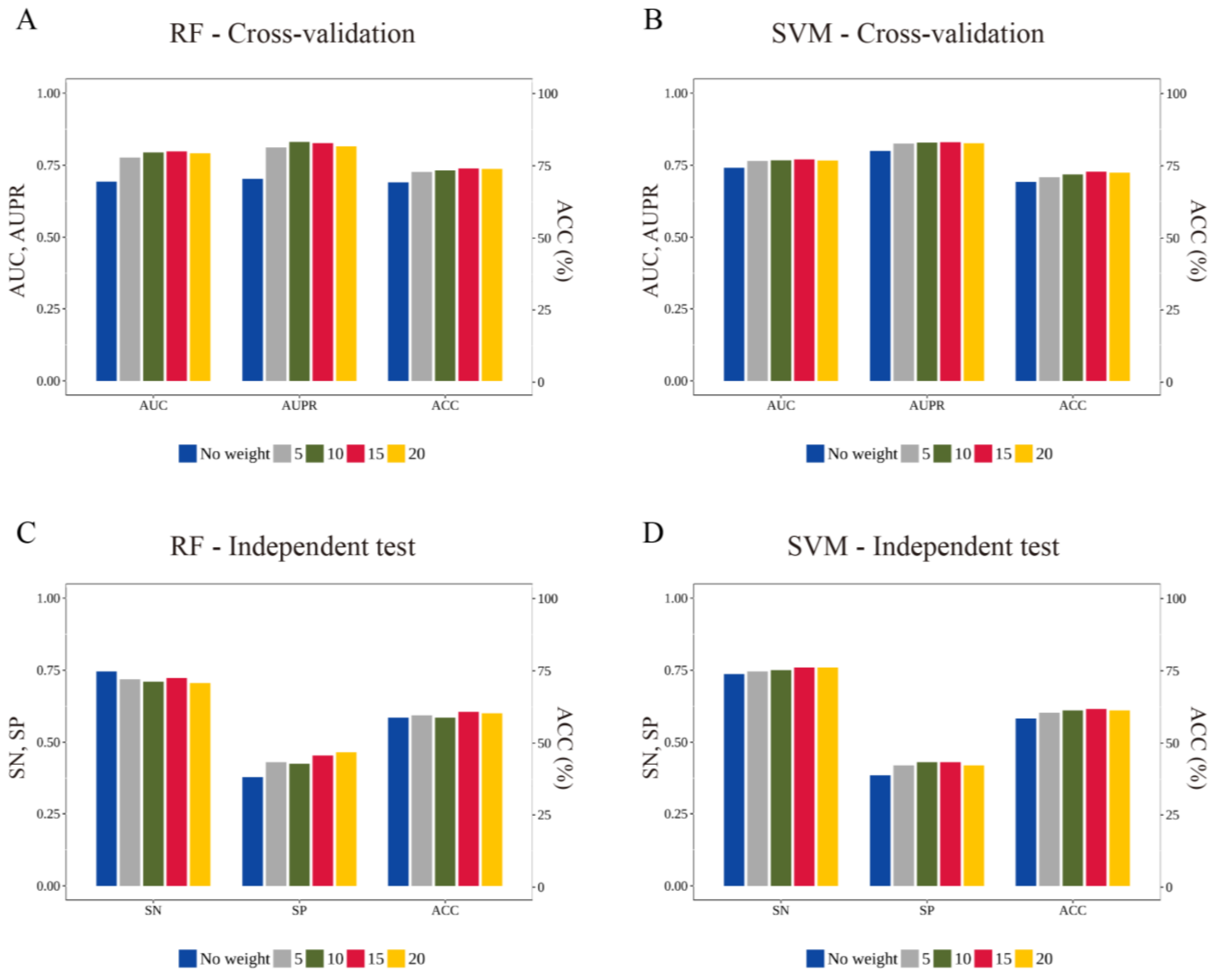

(Threshold: 1.5) **(Threshold: 2)**

A. Performance change in cross-validation by weight values (Random Forest). B. Performance change in cross-validation by weight values (SVM). C. Performance change in independent test by weight values (Random Forest). D. Performance change in independent test by weight values (SVM). The x-axis indicates the multiplied number to selected substructures ranging from 5 to 20.
